# Blood parameters as biomarkers in a *Salmonella* spp. disease model of weaning piglets

**DOI:** 10.1371/journal.pone.0186781

**Published:** 2017-10-26

**Authors:** Emili Barba-Vidal, Victor Fernando Buttow Roll, Edgar Garcia Manzanilla, Carlos Torrente, José Antonio Moreno Muñoz, José Francisco Pérez, Susana María Martín-Orúe

**Affiliations:** 1 Animal Nutrition and Welfare Service, Departament de Ciència Animal i dels Aliments, Universitat Autònoma de Barcelona; Bellaterra, Spain; 2 Federal University of Pelotas, Faculty of Agronomy Eliseu Maciel, Department of Animal Science; Pelotas, RS, Brazil; 3 Pig Development Department, Animal and Grassland Research and Innovation Centre, TEAGASC, The Irish Food and Agriculture Authority, Moorepark; Fermoy, Co. Cork, Ireland; 4 Emergency and Critical Care Service FHCV-UAB, Departament de Medecina i Cirurgia animal, Universitat Autònoma de Barcelona; Bellaterra, Spain; 5 Laboratorios Ordesa S. L.; Barcelona, Spain; INIA, SPAIN

## Abstract

**Background:**

The weaning pig is used as an experimental model to assess the impact of diet on intestinal health. Blood parameters (BP) are considered a useful tool in humans, but there is very scarce information of such indicators in the weaning pig. The objective of the present study is to evaluate the use of different BP as indicators in an experimental model of salmonellosis.

**Methodology:**

Seventy-two 28-day-old piglets were divided into four groups in a 2x2 factorial arrangement, with animals receiving or not a probiotic combination based on *B*. *infantis* IM1® and *B*. *lactis* BPL6 (10^9^ colony forming units (cfu)/d) and orally challenged or not a week later with *Salmonella* Typhimurium (5x10^8^ cfu). Blood samples of one animal per pen (N = 24) were taken four days post-inoculation for the evaluation of different BP using an I-stat® System and of plasmatic concentrations of zinc, iron and copper.

**Principal findings:**

Results reported marginal deficiencies of zinc in piglets at weaning. Moreover, plasmatic zinc, copper and iron presented good correlations with weight gain (r 0.57, r -0.67, r 0.54 respectively; *P <* 0.01). Blood electrolytes (Na^+^, Cl^-^ and K^+^) decreased (*P < 0*.*01*) only when the performance of the animals was seriously compromised and clinical symptoms were more apparent. Acid-base balance parameters such as HCO3^-^, TCO2 and BE_ecf_ significantly correlated with weight gain, but only in the challenged animals (r -0.54, r -0.55, and r -0.51, respectively; *P <* 0.05), suggesting metabolic acidosis depending on *Salmonella* infection. Glucose was affected by the challenge (*P* = 0.040), while Htc and Hgb increased with the challenge and decreased with the probiotic (*P <* 0.05). Furthermore, correlations of Glu, Htc and Hgb with weight gain were observed (*P* < 0.05). Overall, BP could be regarded as simple, useful indexes to assess performance and health of weaning piglets.

## Introduction

The weanling pig has been proposed as a valuable animal model to assess microbiota-health interactions, since pigs exhibit similar syndromes to humans, such as necrotic enterocolitis (NEC) and weanling diarrhea [1]. Moreover, weaning is a critical time in which immature piglets have to face multiple stressors [2,3] and are predisposed to different digestive pathologies frequently related to the overgrowth of opportunistic pathogens like *Salmonella* or *Escherichia coli* [4]. Experimental models of diseases are valuable tools to study in-depth post-weaning syndrome outcomes and to evaluate the potential of preventive or therapeutic treatments, as in the inclusion of probiotics into the diets. In these types of studies, animal performance and physiological parameters such as rectal temperature, fecal scores, fecal shedding of pathogens and a set of different histo-morphologic and immunologic indicators are frequently evaluated. However, very little is known about the potential usefulness of clinical blood parameters as indicators of health status in the weaning piglet. To our knowledge, the use of blood parameters such as plasmatic Zn, Cu, Fe, acid-base or electrolyte balance parameters has not previously been considered as a valuable index in swine.

Portable blood analyzers provide easy access to real-time results within minutes and are used for acid-base and electrolyte balance diagnoses with therapeutic and prognostic implications in humans [5,6] and small animals [7–9]. However, they have only been proposed recently as a useful tool for the early assessment of the health status in nursery pigs [10,11].

Taking this into consideration, the main objective of the present study is to evaluate the potential use of different blood parameters, related to the mineral status, and the acid-base and electrolyte balance in experimental models of gastrointestinal disorders in the weaning piglet. An experimental challenge with *Salmonella* Typhimurium was performed, and pigs received a probiotic combination or not of *Bifidobacterium longum* subsp. *infantis* CECT 7210 (branded as *B*. *infantis* IM1®) [12] and *Bifidobacterium animalis* subsp. *lactis* BPL6. In this context of a controlled model of disease, we hypothesized that *Salmonella* infection would lead to dysregulation of the electrolyte and acid-base balance, and that these effects could be ameliorated by a probiotic supplementation, achieving a wide range of clinical responses. Furthermore, this work aims to contribute to the definition of updated reference values for blood parameters in weaning piglets, considering the scarce information published to-date. Complementary information to this study, regarding the response of the animals in terms of pathogen excretion, fermentation products, ileal histomorphology, plasmatic concentration of pro-inflamatory cytokine TNF-a or acute phase protein Pig-MAP can be found in [13].

## Materials and methods

The experiment was performed at the Experimental Unit of the Universitat Autònoma de Barcelona (UAB) and received prior approval (Permit No. CEAAH1619) from the Animal and Human Experimental Ethical Committee of this Institution. The treatment, management, housing, husbandry and slaughtering conditions strictly conformed to European Union Guidelines [14], and all efforts were made to minimize suffering. The trial was conducted following previously approved biosafety Level 2 procedures, with the appropriate training of the personnel involved.

### Animals, housing and experimental design

Seventy-two Large White x Landrace piglets were selected from a high-sanitary-status breeder farm (gilt multiplier) characterized by a high biosecurity management and a vaccination program including: Porcine Circovirus (PCV), Porcine Proliferative Enteropathy (PPE) and Enzootic Pneumonia (Mycoplasma hyopneumoniae). Farm was also PRRS negative and presented no obvious health challenge at the time of the study. Piglets were weaned at 28 (±2) days of age and brought to the experimental facilities of the UAB. The selected animals were picked from sows which were sero-negative for *Salmonella spp*. and piglets confirmed to be microbiologically negative in feces upon arrival. They were placed in three rooms containing 8 pens (three pigs per pen), according to their body weight. The pens were allocated to four treatment groups following an unbalanced 2 x 2 factorial arrangement (factors being probiotic and *Salmonella* Typhimurium challenge), with eight replicates per treatment for the challenged animals and four replicates for the non-challenged ones. The treatments were: 1) no challenge + no probiotic (NN), 2) no challenge + probiotic (NP), 3) challenged + no probiotic (CN), and 4) challenged + probiotic (CP).

Pigs were maintained under a 14:30h light and 9:30h dark lighting regimen and had *ad libitum* access to water and feed. A pre-starter diet without additives, formulated to satisfy the nutrient requirement standards for pigs [15] containing 18.9% crude protein and 2,470 kcal/kg metabolizable energy (Table 1), was fed to all pigs.

**Table 1:** Dietary composition and nutrient analysis of the experimental diets as-fed basis, g/kg.

**Ingredients**	
Maize	280.8
Wheat	170.0
Barley	150.0
Extruded soybean	122.4
Sweet whey-powder	100.0
Fishmeal LT	50.0
Soybean meal 44	50.0
Whey-powder 50% fat	30.3
Mono-calcium phosphate	21.3
Calcium carbonate	8.2
L-Lysine HCL	4.5
Vitamin-Mineral Premix ^A^	4.0
Sodium chloride	3.0
DL-Methionine 99	2.4
L-Threonine	2.3
L-Tryptophan	0.9
**Chemical composition**	
DM	903.2
Crude Protein	189.3
Neutral detergent fiber	111.6
Ash	74.1
Crude Fat	64.5
Acid-detergent fiber	35.1

^A^ Provided per kilogram of complete diet: 10,200 IU vitamin A, 2,100 IU vitamin D_3_, 39.9 mg vitamin E, 3 mg vitamin K_3_, 2 mg vitamin B_1_, 2.3 mg vitamin B_2_, 3 mg vitamin B_6_, 0.025 mg vitamin B_12_, 20 mg calcium panthotenate, 60 mg nicotinic acid, 0.1 mg biotin, 0.5 mg folic acid, 150 mg Fe, 156 mg Cu, 0.5 mg Co, 120 mg Zn, 49.8 mg Mn, 2 mg I, 0.3 mg Se.

### Probiotic and bacterial inoculation

Pigs received the probiotic treatment orally and individually, in a daily basis using disposable syringes without needle during all the experimental period. In the probiotic group, a daily dosage (10^9^ cfu) of a combination of *Bifidobacterium longum* subsp. *infantis* CECT 7210 (branded as *B*. *infantis* IM1®) [12] and *Bifidobacterium animalis* subsp. *lactis* BPL6 was supplemented in a 2-mL solution, and the control group received the same amount of lactic based sterile carrier as placebo. All treatments were proportioned by Ordesa S.L.

Probiotic concentrations administered were verified by plating shortly after re-suspending the probiotic and after 1 and 2h of bacteria being suspended and left in room temperature. Serial dilutions of the suspension were performed in Man Rogosa Sharpe (MRS) broth (Oxoid; Madrid, Spain) + 0.25% cysteine (Sigma-Aldrich; Madrid, Spain), they were plated in MRS-C agar (Oxoid; Madrid, Spain), incubated at 37°C in anaerobic conditions for 48h and manually counted. Bacterial recounts were always maintained in a logarithm scale of 10^9^ cfu/g (data not shown).

After a 1-week acclimation period, pigs were orally challenged with a 2-mL culture of *Salmonella* Typhimurium (5 x 10^8^ cfu) or received the same amount of sterile media as placebo in the non-challenged group. The *Salmonella* Typhimurium strain was provided by the Veterinary Laboratory of Infectious Diseases (UAB). This strain (ref. 301/99) is a *Salmonella* Typhimurium *var*. *Monophasic* (*formula*: *4*,*5*,*12*:*i*:*-*, *resistance profile*: *ACSSuT-Ge*, *Fagotype*: *U302*) isolated from a salmonellosis outbreak (mainly enteric with sporadic septicemia) in a commercial farm of fattening pigs in Spain. The oral inoculums were prepared by 24h incubation at 37°C in buffered peptone water (Oxoid; Hampshire, UK) and diluted (1:20) with sterile PBS (Sigma-Aldrich; Madrid, Spain) to reach a final concentration of 2.5 x 10^8^ cfu/mL. *Salmonella* concentrations administered were verified by seeding serial dilutions of the inoculum culture in Xylose-Lactose-Tergitol-4 (XLT4) plates (Merck; Madrid, Spain).

### Sampling procedures

Animals were weighed upon arrival, the day of the inoculation and on Day 4 post-inoculation (PI). Moreover, all animals were monitored individually from the day after the challenge (Day 1 PI) up to Day 4 PI: rectal temperature was assessed on Days 1 and 2 PI, and fecal scores were given individually by stimulating animals to defecate (digital stimulation was undertaken if animals did not defecate with handling) on Days 1, 2 and 3 PI. A numeric scale from 1 for normally shaped and solid feces to 4 for severe or bloody diarrhea was used to assess the fecal scores. Fecal samples (5 g) were collected with sterile containers for quantitative *Salmonella* assessment on arrival and on Day 1, 3 and 4 PI. For *Salmonella* bacteria counts, all samples were transferred (1:10) to buffered peptone water. Assessment was made by seeding the serial dilutions 10^−2^ of the samples in Xylose-Lactose-Tergitol-4 (XLT4) plates (Merck; Madrid, Spain).

Blood samples were obtained at Day 4 PI by venipuncture of the cranial vena cava in a sub-sample of 24 pigs (the animal with the intermediate weight of each pen). Ten milliliters of blood were obtained with a 20G needle. A drop was introduced in the portable blood reader and the rest were stored in 10-mL tubes without anticoagulant (Aquisel; Madrid, Spain). Serum was subsequently obtained after centrifugation (2,000 x *g*, 10 min, 15°C) and stored in 1.5-mL aliquots at -20°C until use.

### Blood analysis

Immediately after extraction, whole blood was used for on-site analysis of potassium (K^+^), sodium (Na^+^), chloride (Cl^-^), bicarbonate (HCO_3_^-^), anion Gap (AG), pH, partial pressure of carbon dioxide (pCO_2_), total carbon dioxide (TCO_2_), base excess in the extracellular fluid compartment (BE_ecf_), glucose (Glu), hemoglobin (Hgb) and hematocrit (Htc), using an I-stat® System with a CG8+ cartridge (Abaxis; Union City, California). Bicarbonate (HCO_3_^-^) and BE_ecf_ were calculated by the I-stat® analyzer using the Henderson-Hasselbach formula in conjunction with the Siggaard-Anderson equation and Van Slyke equations, respectively [16]. In all animals, the acid-base analysis was completed by calculating the anion gap (AG = Na^+^ + K^+^–Cl^-^–HCO_3_^-^).

Interpretation of acid-base status was performed using the traditional approach based on the Henderson-Hasselbach equation; the parameters taken into account were pH, pCO_2_, HCO_3_^-^, BE_ecf_, and AG. Acid-base disorders were classified by this method using the criteria given by de Morais and Di Bartola [17]. The four acid-base disturbances described were: respiratory alkalosis or acidosis, and metabolic alkalosis or acidosis. Animals with metabolic acidosis were further characterized considering AG to characterize the presence of high-AG acidosis, that is, acidosis associated with an increased concentration of unmeasured anions.

Serological antibodies of *Salmonella* were tested by ELISA *Salmonella* Herd-check (Idexx; Hoofddorp, 249 Netherlands), and the cut-off for positivity was established at optic density ≥40%.

Serum sub-samples were diluted in a 0.05% p/v EDTA and 0.5% v/v NH_3_ solution to analyze Zinc (Zn), Iron (Fe) and Copper (Cu) by using an Inductively Coupled Plasma Optical Emissions Spectrophotometer (ICP-OES model Optima 4300DV, PerkinElmer Inc.; Waltham, MA, USA) following the procedure described in [18].

### Statistical analysis

The experimental unit was the pig in all variables evaluated individually except for animal performance data, where the pen mean was used. The data were studied by covariance analysis that included the oral challenge and the probiotic as fixed effects and individual body-weight of the pigs at the arrival day as covariate. Moreover, the Pearson correlation procedure was used to study the relationship between blood parameters and weight gain. To evaluate the effects of weight loss on blood parameters, the body-weight gain was categorized into four factors according to quartile range distribution. The Chi-Square test was used to evaluate the association between weight loss and fecal consistency. Odds ratio was calculated to measure association between challenge and febrile response. Multiple-mean comparisons were performed using Tukey’s correction. All data expressed correspond to least squares means (LSmeans) unless otherwise stated. All analyses were carried out using the SAS 9.2 statistical package, and alpha level for determination of significance was *P* < 0.05.

## Results

### Animal performance

Final body weight (BW), average daily feed intake (ADFI) and average daily gain (ADG) was reduced by the oral challenge (*P =* 0.024, *P =* 0.006 and *P* < 0.001, respectively), which even caused weight losses during the post-inoculation period in some animals (See Table 2).

**Table 2:** Effect of the experimental treatments on growth performance.

	Treatments ^A^					P-value		
	NN	NP	CN	CP	RSD ^B^	Challenge ^C^	Probiotic ^D^	Interaction
**Average BW**^**E**^ **(kg)**								
**Initial**	7.60	7.85	7.90	7.51	*0*.*559*	*0*.*929*	*0*.*770*	*0*.*195*
**Final**	8.87	9.06	8.05	8.26	*0*.*763*	*0*.*024*	*0*.*543*	*0*.*977*
**ADFI** ^**F**^**(g/day)**								
**Pre-inoculation**^**G**^	273	250	259	306	*55*.*9*	*0*.*391*	*0*.*611*	*0*.*164*
**Post-inoculation**^**H**^	395	398	218	276	*112*.*3*	*0*.*006*	*0*.*536*	*0*.*569*
**ADG (g/day)**^**I**^								
**Pre-inoculation**	78	58	53	119	*50*.*5*	*0*.*417*	*0*.*303*	*0*.*063*
**Post-inoculation**	240	268	-71	-25	*119*.*3*	*<0*.*001*	*0*.*484*	*0*.*858*

^A^ Treatments: NN (non-challenged + no probiotic), NP (non-challenged + probiotic), CN (challenged + no probiotic) and CP (challenged + probiotic). Challenged groups n = 8; non-challenged groups n = 4.

^B^ Residual Standard Deviation.

^C^ Challenge: main effect of the *Salmonella* Typhimurium inoculation (5 x 10^8^cfu).

^D^ Probiotic: main effect of a daily dosage (10^9^cfu) of a combination of *B*. *infantis* IM1® and *B*. *lactis* BPL6. ^E^ Body weight (BW) (kg) at experimental Days 0 and 11 (4 days post-inoculation). ^F^ Average daily feed intake (ADFI) (g/day) ^G^ Pre-inoculation (0–7th day before the challenge). ^H^ Post-inoculation period (0–4th day after the challenge). ^I^ Average daily gain (ADG) (g/day).

### Clinical parameters and pathogen seeding

In general, animals showed a good health status at the beginning of the experiment before the challenge. During the PI period, two deaths were registered in the challenged groups; one from the CN group on Day 4 PI and one from the CP group on Day 3 PI. Necropsy was performed on the dead animals and both of them presented fibrino-hemorragic gastritis and acute diffuse fibrinous enteric-tiflo-colitis, lesions normally associated with infection of *Salmonella* Typhimurium in pigs [19]. No antibiotic treatment was administered to any of the animals in the trial.

None of the animals seeded *Salmonella* in feces on arrival, and only challenged groups seeded *Salmonella* in countable concentrations (>10^3^ cfu/g) at Day 1 PI (87.5% for CN and 100% for CP groups), Day 3 and 4 PI (100% for CN and CP groups) after the challenge. Serological analysis confirmed that animals had not been exposed to *Salmonella* before the day of inoculation, all animals being analyzed as seronegative along the whole trial.

The challenge was associated with a febrile (>40.0°C) response (odds ratio 11.67, 95% confidence interval 1.14–119.542; *P* = 0.039), with challenged animals presenting higher mean rectal temperatures 24 hours PI than did the non-challenged animals (40.0°C *vs*. 39.3°C, *P* = 0.010). No changes were associated with the probiotic administration at 24 hours.

**Fig 1:**
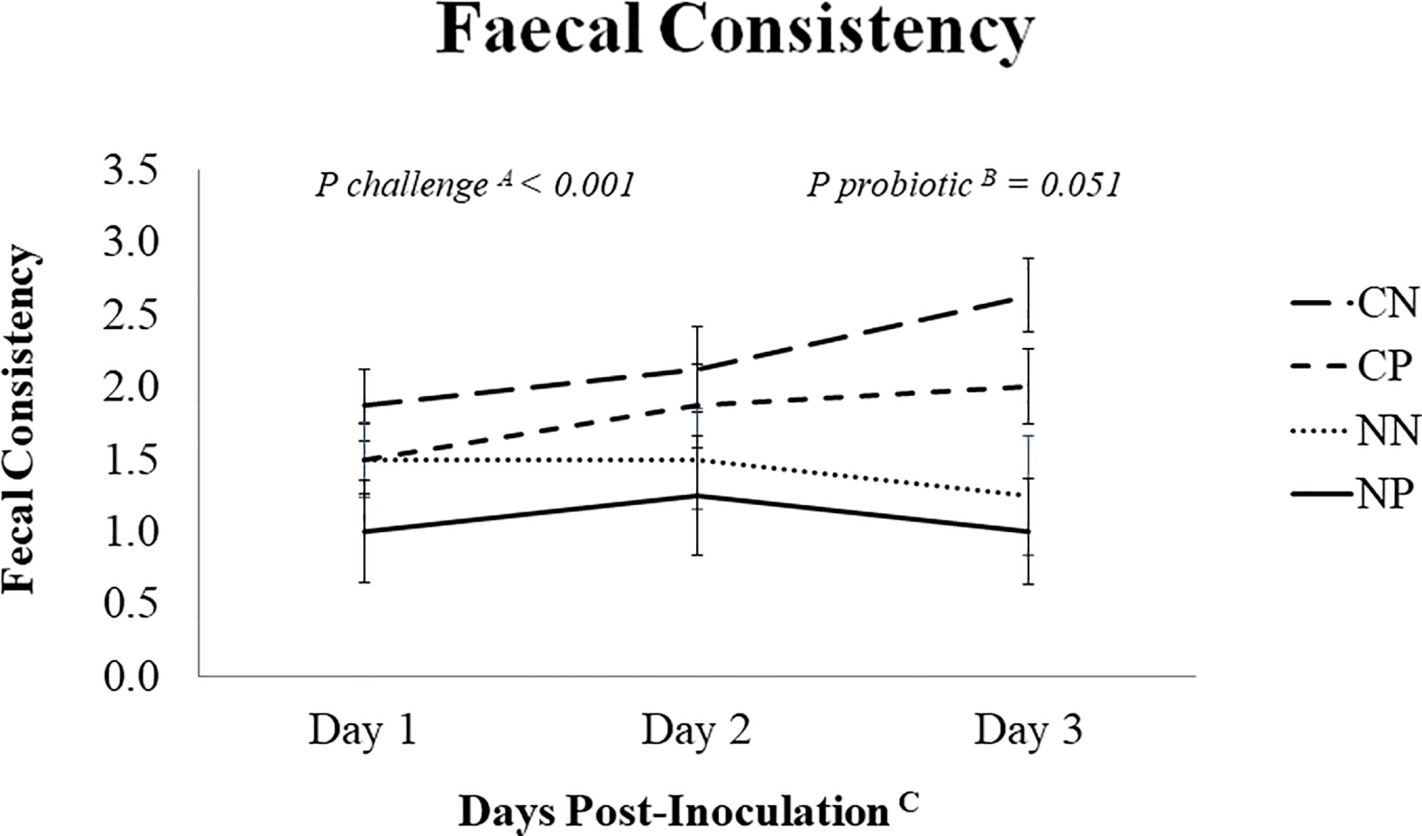
Evolution of the mean fecal scores in the different experimental groups during the post-inoculation period. Treatments: CN (challenged + no probiotic), CP (challenged + probiotic), NN (non-challenged + no probiotic) and NP (non-challenged + probiotic) (challenged groups n = 8; non-challenged groups n = 4). Values represented are LSmeans with their standard errors. ^A^ Challenge: main effect of the *Salmonella* Typhimurium inoculation (5 x 10^8^cfu). ^B^ Probiotic: main effect of a daily dosage (10^9^cfu) of a combination of *B*. *infantis* IM1^®^ and *B*. *lactis* BPL6. ^C^ Days after the challenge with *Salmonella*.

However, at 48 h the probiotic tended to reduce rectal temperature in challenged animals, when they presented a similar temperature to the non-challenged (39.8°C for CN, 39.2°C for CP, 39.1°C for NN, 39.0°C for NP; *P* challenge x probiotic = 0.095). The oral inoculation of the pathogen promoted moderate diarrhea in most of the animals, with significant increases in the fecal score (See Fig 1). Administration of the probiotic showed a trend to improve the fecal consistency with decreases in the fecal score in both challenged and non-challenged animals (1.8 ± 0.13 for control *vs*. 1.4 ± 0.13 for probiotic; *P =* 0.051 for Days 1 to 3 PI). In general, diarrhea was mild, but severity of diarrhea was greater in animals with a lower weight, as was demonstrated when analyzing results of fecal scores by animal weight-gain quartiles (average fecal scores 2.48, 1.96, 1.62 and 1.24 for 1^st^, 2^nd^, 3^rd^ and 4^th^ quartile; *P* < 0.001).

Other important effects of the *Salmonella* challenge at intestinal level were a significant reduction in colonic fermentation with lower concentrations SCFA (96 vs 137 mmol/kg, *P* = 0.03), and an impaired intestinal architecture with reductions in villous height (188 vs. 258 μm, *P* = 0.03) and increases in the number of mitosis in cript cells (0.39 vs. 0.26 n° cells/100 μm). When analyzing plasmatic pro-inflamatory citokine TNF-α it was significantly increased by the challenge (155 vs. 83 pg/ml, *P* < 0.001) and also the acute phase protein Pig-MAP (2.85 vs 1.07 mg/ml, *P* = 0.03). The probiotic treatment was not able to revert any of these effects at day 4 PI. More detailed information can be found in Barba-Vidal et al. (2017)

### Blood parameters

Changes in blood parameters due to the *Salmonella* challenge and probiotic treatment are shown in Table 3.

**Table 3:** Change in blood parameters in pigs orally challenged or not with *Salmonella* and receiving a probiotic mixture or not.

	Treatments ^A^					P-Value		
	NN	NP	CN	CP	RSD ^B^	Challenge ^C^	Probiotic ^D^	Interaction
**Zn (mg/L)**	0.72	0.64	0.51	0.63	*0*.*119*	*0*.*049*	*0*.*801*	*0*.*093*
**Cu (mg/L)**	1.33	1.41	1.72	1.76	*0*.*223*	*0*.*001*	*0*.*540*	*0*.*863*
**Fe (mg/L)**	7.13	5.37	3.68	3.35	*3*.*91*	*0*.*127*	*0*.*550*	*0*.*679*
**Na**^**+**^ **(mM)**	138.6	140.9	130.5	133.4	*9*.*308*	*0*.*076*	*0*.*551*	*0*.*945*
**K**^**+**^ **(mM)**	5.43	5.99	4.83	5.8	*0*.*728*	*0*.*233*	*0*.*034*	*0*.*540*
**Cl**^**-**^ **(mM)**	105.3	106.3	101.2	105.2	*0*.*76*	*0*.*521*	*0*.*553*	*0*.*691*
**HCO**_**3**_^**-**^ **(mM)**	25.6	22.3	24.5	19.3	*5*.*12*	*0*.*446*	*0*.*110*	*0*.*712*
**AG (mM)**	13.6	17.5	14.5	14.8	*2*.*92*	*0*.*544*	*0*.*175*	*0*.*244*
**pH**	7.44	7.40	7.41	7.43	*0*.*138*	*0*.*990*	*0*.*816*	*0*.*673*
**TCO**_**2**_ **(mM)**	26.7	23.2	25.7	20.1	*5*.*39*	*0*.*472*	*0*.*112*	*0*.*703*
**pCO**_**2**_ **(mmHg)**	37.5	35.0	39.4	29.5	*11*.*45*	*0*.*758*	*0*.*291*	*0*.*517*
**BE**_**ecf**_ **(mM)**	1.36	-2.47	-0.11	-4.96	*6*.*414*	*0*.*555*	*0*.*189*	*0*.*874*
**Glu (mg/dL)**	114.7	110.4	90.9	95.4	*18*.*27*	*0*.*040*	*0*.*995*	*0*.*612*
**Htc (%)**	18.6	10.8	24.9	20.9	*5*.*32*	*0*.*005*	*0*.*030*	*0*.*459*
**Hgb (g/dL)**	6.33	3.69	8.44	7.10	*1*.*814*	*0*.*006*	0.030	0.450

Blood parameters: Zinc (Zn), copper (Cu), iron (Fe), sodium (Na^+^), potassium (K^+^), chloride (Cl^-^), bicarbonate (HCO_3_^-^), anion gap (AG), pH, total carbon dioxide (TCO_2_), partial pressure of carbon dioxide (pCO_2_), base excess in the extracellular fluid compartment (BE_ecf_), glucose (Glu), hematocrit (Htc) and hemoglobin (Hgb). ^A^Treatments: NN (non-challenged + no probiotic), NP (non-challenged + probiotic), CN (challenged + no probiotic) and CP (challenged + probiotic). Challenged groups n = 8; non-challenged groups n = 4.

^B^ Residual Standard Deviation.

^C^ Challenge: main effect of the *Salmonella* Typhimurium inoculation (5 x 10^8^cfu).

^D^ Probiotic: main effect of a daily dosage (10^9^ cfu) of a combination of *B*. *infantis* IM1^®^ and *B*. *lactis* BPL6.

The oral challenge promoted a decrease in plasmatic Zn (0.69 *vs*. 0.57 mg/L; *P =* 0.049), which tended to be more important in the CN group (*P* challenge x probiotic = 0.093), and an increase in Cu (1.37 *vs*. 1.74 mg/L; *P* < 0.001). A tendency to decrease Na^+^ concentrations was detected (139.7 mM *vs*. 131.9 mM; *P* = 0.076) in the measured electrolytes concentrations in response to the oral challenge. In relation to blood biochemistry, the challenge caused a decrease in Glu (112.6 mg/dL *vs*. 93.1 mg/dL; *P* = 0.040) together with greater values of Htc and Hgb (14.7% *vs*. 22.9%; *P* = 0.005 for Htc and 5.01 g/dL *vs*. 7.77 g/dL; *P* = 0.006 for Hgb).

The probiotic treatment increased blood K^+^ concentrations (5.13 mM *vs*. 5.90 mM; *P* = 0.034) and decreased values of Htc and Hgb (21.71% *vs*. 15.85% for Htc and 7.39% *vs*. 5.4% for Hgb).

Weight-loss values were correlated with the different blood parameters tested to discover the strength of the association between these two factors, regardless of the experimental treatments (Table 4).

**Table 4:** Pearson correlation analysis between weight gain and blood parameters in weaning piglets orally challenged or not with *Salmonella* Typhimurium.

		Zn	Cu	Fe	Na^+^	K^+^	Cl^-^	HCO_3_^-^	AG	pH	TCO2	pCO2	BE_ecf_	Glu	Htc	Hgb
**All Animals (n = 24)**	**r**	0.57	-0.67	0.54	0.67	0.40	0.52	-0.27	0.18	-0.17	-0.28	-0.07	-0.29	0.47	-0.42	-0.42
	***P-*value**	*0*.*006*	*0*.*001*	*0*.*008*	*0*.*001*	*0*.*055*	*0*.*012*	*0*.*231*	*0*.*436*	*0*.*444*	*0*.*213*	*0*.*755*	*0*.*195*	*0*.*024*	*0*.*048*	*0*.*048*
**Non- challenged (n = 8)**	**r**	0.74	-0.58	0.61	-0.25	0.16	0.01	0.11	-0.18	-0.19	0.11	0.53	0.02	0.67	0.22	0.23
	***P-*value**	*0*.*037*	*0*.*128*	*0*.*106*	*0*.*558*	*0*.*707*	*0*.*995*	*0*.*838*	*0*.*731*	*0*.*714*	*0*.*837*	*0*.*275*	*0*.*974*	*0*.*102*	*0*.*631*	*0*.*622*
**Challenged (n = 16)**	**r**	0.38	-0.56	0.374	0.71	0.44	0.65	-0.54	0.33	-0.15	-0.55	-0.25	-0.51	0.31	-0.42	-0.42
	***P-*value**	*0*.*186*	*0*.*023*	*0*.*169*	*0*.*002*	*0*.*089*	*0*.*008*	*0*.*031*	*0*.*235*	*0*.*579*	*0*.*028*	*0*.*345*	*0*.*044*	*0*.*244*	*0*.*104*	*0*.*101*

Blood parameters: Zinc (Zn), copper (Cu), iron (Fe), sodium (Na^+^), potassium (K^+^), chloride (Cl^-^), bicarbonate (HCO_3_^-^), anion gap (AG), pH, total carbon dioxide (TCO_2_), partial pressure of carbon dioxide (pCO_2_), base excess in the extracellular fluid compartment (BE_ecf_), glucose (Glu), hematocrit (Htc) and hemoglobin (Hgb). Weight gains (kg) were calculated by the difference between body weights registered at Experimental Days 0 and 11 (4 days post-inoculation).

Many parameters showed strong correlations with weight gain. Particularly significant, positive correlations were found for Zn, Fe, Na^+^, K^+^, Cl^-^ and Glu; whereas Cu, Htc and Hgb showed negative correlations. Parameters related to the electrolyte or the acid-base balance, such as HCO_3_^-^, AG, pH, TCO_2_, pCO_2_ or BE_ecf_ did not show any remarkable relation to weight gains. When considering exclusively non-challenged animals, a high, positive correlation to weight gain was seen with Zn concentrations. On the other hand, in challenged animals, positive correlations were seen with electrolytes Na^+^, K^+^ and Cl^-^, while negative correlations were seen with Cu, HCO_3_^-^, TCO_2_ and BE_ecf._

Finally, mean values of blood parameters for the different quartiles, considering the weight gain of the animals, are represented in Table 5. Zinc, copper and iron responded proportionally to the weight gain, while Na^+^, K^+^, Cl^-^ only showed significantly lower values in the group of animals with the lowest weights.

**Table 5:** Comparisons between mean blood parameters in groups of pigs (n = 6) stratified by body-weight gain.

	Quartile Distribution ^A^					
	1st Quartile	2nd Quartile	3rd Quartile	4th Quartile	RSD ^B^	*P-*value
**Zn (mg/L)**	0.52 ^b^	0.53 ^b^	0.66 ^ab^	0.71 ^a^	*0*.*113*	*0*.*025*
**Cu (mg/L)**	1.79 ^a^	1.71 ^a^	1.64 ^ab^	1.32 ^b^	*0*.*218*	*0*.*007*
**Fe (mg/L)**	2.08 ^b^	1.79 ^b^	7.07 ^a^	7.45 ^a^	*3*.*024*	*0*.*005*
**Na (mM)**	123.8 ^b^	137.5 ^a^	137.6 ^a^	139.3 ^a^	*7*.*27*	*0*.*005*
**K (mM)**	4.55 ^b^	5.72 ^a^	6.13 ^a^	5.40 ^ab^	*0*.*593*	*0*.*001*
**Cl (mM)**	94.0 ^b^	108.5^a^	106.3 ^a^	106.4 ^a^	*5*.*40*	*0*.*002*
**HCO**_**3**_^**-**^ **(mM)**	25.4	20.2	20.3	23.7	*5*.*16*	*0*.*281*
**AG (mM)**	14.0	14.8	16.2	14.6	*3*.*00*	*0*.*699*
**pH**	7.45	7.43	7.37	7.43	*0*.*135*	*0*.*775*
**TCo**_**2**_ **(mM)**	26.5	21.3	21.2	24.6	*5*.*46*	*0*.*311*
**pCo**_**2**_ **(mmHg)**	37.4	32.0	34.4	35.9	*12*.*06*	*0*.*884*
**BE (mM)**	1.50	-4.00	-5.00	-0.80	*6*.*184*	*0*.*303*
**Glu (mg/dL)**	90.7	92.8	99.8	115.2	*17*.*43*	*0*.*127*
**Htc (%)**	23.7	20.5	16.3	20.4	*6*.*47*	*0*.*305*
**Hgb (g/dL)**	8.05	6.96	5.57	6.94	*2*.*202*	*0*.*310*

Blood parameters: Zinc (Zn), copper (Cu), iron (Fe), sodium (Na^+^), potassium (K^+^), chloride (Cl^-^), bicarbonate (HCO_3_^-^), anion gap (AG), pH, total carbon dioxide (TCO_2_), partial pressure of carbon dioxide (pCO_2_), base excess in the extracellular fluid compartment (BE_ecf_), glucose (Glu), hematocrit (Htc) and hemoglobin (Hgb). ^A^Weight gains (kg) were calculated by the difference between body weights registered on Experimental Days 0 and 11 (4 days post-inoculation) and were stratified as following: 1st Quartile (-1.18 kg-0.34 kg), 2nd Quartile (0.4 kg-1.16 kg), 3rd Quartile (1.26 kg-1.66 kg) and 4th Quartile (1.8 kg-3.22 kg). ^B^ Residual Standard Deviation. Blood parameter values are means. Means with different letters are significantly different, (*P* < 0.05) by Tukey- test.

## Discussion

The main objective of the present study was to evaluate the potential use of different blood parameters related to the mineral status and the acid-base and electrolyte balance as health indicators in piglets experimentally challenged with *Salmonella* Typhimurium and receiving a probiotic combination or not of *B*. *infantis* IM1^®^ and *B*. *lactis* BPL6.

The challenge with *Salmonella* Typhimurium succeeded in causing an acute dysregulation of gut function, with diarrhea and the affection of several clinical parameters. On the other hand, the probiotic provoked moderate benefits in several parameters, allowing for the achievement of a wide range of controlled clinical responses.

### Plasmatic micro-minerals

In relation to micro-mineral concentrations, the experimental challenge promoted a decrease in plasmatic Zn. In humans, it has been reported how pro-inflammatory cytokines during infectious diseases regulate changes in Zn hepatic reservoirs in liver cells leading to hypozincemia [20]. This effect could partly explain the results of the reduction of Zn in pigs challenged with *Salmonella* spp., ameliorated in the challenged group receiving probiotic with similar Zn concentrations to the non-challenged groups. However, normal serum Zn concentrations are reported to be within the range of 0.7 mg/L and 1.5 mg/L and serum Zn concentrations associated with marginal status within the range of 0.4 mg/L and 0.8 mg/L [21]. Therefore, values reported in this work would suggest that all of our piglets, challenged and non-challenged ones, showed a marginal deficiency of Zn. This result is in consonance with other authors reporting Zn status of piglets at weaning [18,22].

Some authors have described correlations between plasmatic Zn concentrations and weight gains [23,24] and it has been postulated that one of the first signs of mild Zn deficiency in growing animals is reduced growth [25]. To our knowledge, this is one of the few studies in which such a relationship is seen in weanlings receiving nutritional concentrations of Zn in the diet. Different reasons could explain this relationship between plasmatic Zn and growth in weanlings, such as multiple correlated conditions like infection, trauma, stress, or dietary deficiency, which can reduce both plasmatic concentrations of Zn and growth [18,26]. Nevertheless, in our study this relationship was stronger when only the non-challenged animals were included in the analysis. This fact would suggest that the relationship is not just explained by concomitant effects of stressors on plasmatic Zn and growth concentrations, but also due to the growth retardation described by other authors as a way to preserve body Zn [23,24]. Supporting this, it has been reported that the inclusion of therapeutic levels of 2000–2500 ppm can restore plasma Zn concentrations and improve performance in comparison to animals receiving the same diet with only nutritional concentrations of Zn [18,27,28]. Although the exact mechanism has not been described yet, it may be hormone-mediated, as [29] found a growth stimulating effect mediated by insulin-like growth factor I in undernourished children supplemented with Zn.

On the other hand, challenged groups also presented an increase in Cu concentrations. It has been reported that stress can promote a sustained increase in plasma Cu concentrations, which could be explained by the binding of Cu to caeruloplasmin, a widely recognized acute-phase protein [30]. Additionally, Cu showed an inverse correlation to weight gain, increasing as the animals reduced growth. The reason for this decrease in the animals with better performance is not clear. Carlson (2007) reported decreases in the plasmatic Cu concentration during the first 2 weeks after weaning. In addition, Zn and Cu are physically and chemically similar elements that could act antagonistically in the body. Oral administration of Zn supplements usually promote a decrease in the plasmatic concentrations of Cu due to a decrease in Cu absorption [30]. The complementary responses in Cu and Zn could be the result of a different binding to organic proteins. Metallothioneins (MT) are known to be induced by exposure to heavy metal cations [31], and specifically the expression level of MT1 in jejunum is greater with high dietary concentrations of Zn in piglets [32]. These MT bind metals in the mucosal surface of enterocytes, forming a block that prevents their movement through the cell, thereby limiting absorption of metals. However, in our case, as Zn concentrations are on a nutritional level, it seems improbable that the low plasmatic Cu in bigger animals was a result of interactions in intestinal absorption. In a practical sense, good correlations of Zn and Cu to weight gains could turn them into good descriptors of the performance of the animals at the end of the nursery phase.

Regarding Fe, animals received a 200 mg intramuscular iron injection on their first week of life, a common procedure routinely undertaken in pig farms to prevent iron deficiency [33]. This could be the reason why values found were above the normal range described in the literature [34,35]. Even that when analyzing our results it is interesting to see that challenged animals showed a trend (*P* = 0.13) for lower Fe concentrations. Lower plasma iron concentrations are frequent after infections [36]. An important strategy of mammalian antimicrobial defense is based on depriving pathogens of this essential nutrient. Under the influence of cytokines, macrophages infected by intracellular microbes inhibit their multiplication by moving iron from the phagosomes to cytoplasmic ferritin and inflammation-regulated proteins chelate iron, trap siderophores, and transport iron to alter its tissue distribution; thus lowering the extracellular iron concentration [36,37]. On the other hand, Fe presented a good correlation with weight gains when all animals were included in the analysis; which was confirmed with higher Fe concentrations in animals in third and fourth quartile of weight gain. This result was not expected because it has been described that larger piglets at this age present a higher risk of Fe deficiency [38]. However, it is well known that health effects and zootechnical effects are closely related [39] and in our case infected animals were the ones with lowest weight gains. Hence, lower iron levels observed in animals with the lowest weight gains can be explained by the innate immune system of these restricting iron availability to *Salmonella* [36,37].

### Blood electrolytes

The only blood electrolyte significantly altered by the experimental treatments was the K^+^ concentration. The probiotic administration enhanced K^+^ blood concentration, restoring the concentration of K^+^ to the concentrations registered in the non-challenged group. This could suggest a relative improvement of animals receiving probiotics and could be related to the beneficial effects observed by the probiotic treatment in fecal consistency. In relation to the challenge effects, we suspect that too mild symptoms, acuteness of diarrhea and inter-individual variability promoted by our oral challenge could explain the scarce differences found, considering that changes depend on the cause of diarrhea, its severity and chronicity [40].

When related to body-weight gains, blood concentrations of Na^+^, K^+^ and Cl^-^ were significantly reduced only in those animals with the lowest gains, suggesting a breaking-point reached only by those animals seriously compromised and probably due to the loss of these electrolytes through acute diarrhea. These could be due to the fact that they are key electrolytes with essential functions in the body. Sodium and chloride are the main extracellular cation and anion, respectively, in the body and influence the electrolytic balance and acid-base status of animals. As for potassium, it is implicated in electrolyte balance, neuromuscular function and also acts as the monovalent cation to balance intracellular anions, as part of the Na^+^/K^+^ pump physiological mechanism [41]. The mammal organism has different ways to maintain homeostasis of essential nutrients, and probably only when the health status is seriously compromised, such as in the first quartile animals, responses in these indexes can be find.

### Acid-base balance parameters

The anion gap evaluates the difference between the measured cations and anions in the blood. Around two-thirds of the AG comes from the negative charge of serum proteins, while one-third is due the accumulation of phosphate and strong anions in serum, such as L-lactate, sulfate, and anions associated with uremia [42]. Thus, a high-AG metabolic acidosis is supposed to be formed by an acid that does not have chloride as its anion, and a normal-AG metabolic acidosis is accompanied by an equal increase in the plasma chloride concentration to balance the decrease in plasma HCO_3_^-^ concentration [9]. Our results were within the biologically normal range for healthy piglets (12–23mmol/L) [17] and were not significantly different between treatments, suggesting an absence of disturbance caused by the *Salmonella* challenge or the probiotics.

Respiratory acidosis and alkalosis occur when pCO_2_ concentrations are above or below a reference range. Similarly, metabolic acidosis or alkalosis happens when HCO_3_^-^, and/or BE_ecf_ values are, respectively, below or above a reference range. Blood BE_ecf_ values near to zero are desirable [43], as they reflect the maintenance of the required acid-base balance for better performance [44]. Alternatively, metabolic acidosis is characterized by reduced blood pH as a result of the accumulation of non-volatile acids or loss of serum bicarbonate [45]. In our experiment, results for pH, pCO_2_, HCO_3_^-^ and BE_ecf_ were not significantly different between treatments, suggesting that our challenge did not reach the level to disturb the acid-base balance of the animals. However, some acute phase response changes may occur from 12 to 48h post-challenge with *Salmonella* [46], so with our present data we cannot discard that some of the non-significant parameters may have been different if earlier or more frequent blood samples had been collected. This possibility should definitely be explored in future studies.

Remarkably, HCO_3_^-^ concentration was not related to weight gain when all animals were considered in the analysis. However, a negative correlation was found when the challenged group was analyzed separately. This could suggest that although the appearance of metabolic acidosis was not found to be directly related to the *Salmonella* challenge, it could still be present in some of the challenged animals, depending on the level of sickness. Impaired inflammatory response and anorexia in most critically ill animals could be causing these results [47,48]. Supporting this idea, other acid-base parameters (TCO2 and BE_ecf_) were not correlated to weight gain when we consider all of the animals, but significant negative correlations can be observed when we only consider the challenged group.

### Chemical biochemistry

Regarding the chemical biochemistry, we could detect differences related to the oral challenge on Glu, Htc and Hgb concentrations. Glucose concentrations decreased significantly with the challenge. We hypothesize this fact could be due to reduced feed intake also reported with the challenge or a greater blood glucose uptake when the metabolism is responding against the *Salmonella* infection [49,50]. However, it could also be a secondary hypoglycemia in response to the prominent hyperglycemia expected shortly after the challenge due to sepsis [51] and induced by increased cortisol levels [46,52]. A positive correlation was also seen with Glu in relation to weight gain. When assessing these differences by quartiles, a linear increase in Glu concentrations was seen together with weight gains, although these differences were only numerical when studied by quartiles.

Regarding Htc and Hgb, values detected were in the low range than would be expected for piglets at this age [34,53]. However, they were the only two blood parameters that showed significant differences related to both experimental treatments, the challenge and the probiotic. Values of Htc and Hgb were greater in challenged pigs than in control pigs. Other authors have also described how blood Glu decreases and Hct increases during disease in pigs, suggesting a possible dehydration and malnutrition compared to healthy animals [10]. Nevertheless, it has been reported that I-stat® may underestimate Htc and Hgb with hypo-proteinemia, which could be happening in challenged animals with more severe diarrhea [54,55]. This possibility must be considered because proteinemia was not evaluated in our study.

Regarding the changes observed with the administration of probiotics, as far as we know this is the first reported work that uses these indexes to evaluate the efficiency of a probiotic therapy. Similarly to probiotic effects observed in K^+^, decreases observed in Htc and Hgb values with the probiotic treatment could suggest an improvement in the response of the animals against the *Salmonella* challenge, reducing the severity of diarrhea. In addition, the probiotic treatment was also able to decrease Htc and Hgb values in the non-challenged group. Although these last animals did not receive an oral *Salmonella* dose, it is widely known that the weaning itself is a powerful stressor that causes anorexia, growth stasis and intestinal transit dysbiosis [2,3,56].

Furthermore, Htc and Hgb showed significant negative correlations with weight gains, although the difference was only numeric. As discussed before, decreases in Htc and Hgb concentrations could be reflecting the level of dehydration, considering that both indexes are based on whole blood and are therefore dependent on plasma volume. In this regard, Balsbaugh [57] described an increase in the Htc values in pigs in which diarrhea was experimentally-induced, concomitant with body-weight loss. However, our results show that although having a significant correlation with weight gain, this relationship is not strictly linear. We suspect these results may be influenced by an underestimation of these values by the I-stat^®^ in the more severely affected animals [54,55]. In this case, Htc and Hgb data measured by I-stat^®^ should be used with precaution because they may fail to detect animals in worse conditions, in contrast to blood concentrations of Na^+^, K^+^ and Cl^-^, which were significantly different in animals of the lowest quartiles.

## Conclusions

Results of this work support previously reported data that suggest marginal deficiencies of Zn in piglets at weaning and an inverse correlation to Cu concentrations. Moreover, blood parameters have been demonstrated to be good descriptors of pig performance as an index of weanling health status. Micro-minerals Zn, Cu and Fe were well correlated with piglets weight gain. Blood electrolytes (Na^+^, Cl^-^ and K^+^) and acid-base indexes (HCO_3_^-^, TCO2 and BE_ecf_) may enable to detect the most distressed animals. Finally, Htc and Hgb were able to respond to both the challenge and the probiotic treatment suggesting to be good indexes to test the efficacy of in-feed treatments at weaning. However, they may not be useful to identify the most severely affected animals.

## Supporting information

S1 File NC3Rs ARRIVE guidelines. (PDF)

S2 File Raw data. (XLSX)
